# What Keeps Faculty Coming Back? Factors Associated with Continued Pursuit of Faculty Development

**DOI:** 10.1007/s40670-025-02422-8

**Published:** 2025-06-21

**Authors:** David A. Lindholm, Jessica Servey, Rhiana Saunders, Thomas McFate, Salvatore Sidoti, Bizualem Zelelew, Dana Blyth, Diane Hale, Gayle Haischer-Rollo

**Affiliations:** 1 Uniformed Services University of the Health Sciences, Bethesda, MD USA; 2 Brooke Army Medical Center, Joint Base San Antonio-Fort Sam, Houston, TX USA; 3 Walter Reed National Military Medical Center, Bethesda, MD USA

**Keywords:** Faculty development, Professional development, Attendance, Medical school, Teaching hospital, Clinician-educator

## Abstract

**Introduction:**

Faculty development benefits clinician-educators, yet little is known about what influences clinical faculty to pursue development as educators. To optimize faculty development attendance at a federal medical school with geographically dispersed teaching hospitals, we assessed how demographic and workshop factors impacted attendance by comparing physician faculty who attended faculty development on a single day with those returning any future day.

**Materials and Methods:**

We conducted a retrospective review of our faculty development website database for attendee, instructor, and workshop factors among physicians attending their first workshop in 2014–2022. We employed a multivariate model to determine a relative return ratio (RRR) and evaluate independent factors for return.

**Results:**

Of 3,213 attendees, 2,204 (68.6%) returned on any future day, with 966/2,204 (43.8%) returning within one week. Surgical faculty were 11% less likely to return than medical faculty (RRR 0.89; 95% CI 0.84 to 0.95), an effect heightened when both attendee and first instructor were surgeons. Workshops delivered by community-based instructors demonstrated decreased return compared to those delivered by university-based instructors (RRR 0.95; 95% CI 0.90 to 0.99). Attendees whose first workshop occurred at large teaching hospitals with an instructor traveling from main campus were more likely to return than in other contexts. Day, month, and time of workshops also correlated with return.

**Discussion:**

Our study suggests that demographic and workshop factors can influence clinician-educators to continue attending faculty development. We encourage faculty development leaders to evaluate attendance patterns vis-à-viz van Bruggen’s 4Cs of competence, context, community, and career to meet the dynamic needs of their faculty.

**Supplementary Information:**

The online version contains supplementary material available at 10.1007/s40670-025-02422-8.

## Introduction

Clinician-educators are recognized as skilled teachers, yet clinical expertise and pedagogical skills are often conflated. Medical education accrediting agencies now mandate faculty development to strengthen teaching skills. In the United States, both the Liaison Committee on Medical Education (LCME) [1] and the Accreditation Council for Graduate Medical Education (ACGME) [2, 3] require faculty development programs, and recent clinician-educator milestones endorsed by multiple agencies [3] align with prior calls for a standardized educator skill set [4–6]. Accordingly, diverse faculty development initiatives have emerged, demonstrating positive impacts at individual and organizational levels [7–10]. However, there remains a gap in the literature in understanding who attends faculty development and returns for more [9].

Few medical education studies address factors influencing faculty development attendance. One study found no correlation between attendance and academic rank [11]. Others suggest that timing of classes may impact attendance [12] and that an initial positive experience [13] or a curriculum emphasizing reflection [14] may enhance long-term engagement. Institutional factors, such as protected time for faculty development and the percentage of research responsibilities, further influence attendance [14]. Multiple barriers to medical faculty attendance have been identified, including time constraints, lack of clear clinician-educator career advancement pathways, demanding clinical workload, and lack of institutional leadership support [12, 15].

Studies assessing faculty development attendance in other health professions are also limited but have identified several catalysts for attendance: internal motivation, the importance of the content and personal educational needs, policy, and organizational conditions and support [16, 17]. In one study, the presence of a formal policy increased the attendance of psychologists, but no specific attendance was documented [18]. In another study, 80% of surveyed nurses attended at least one professional development session over the prior twelve months [19].

Literature on faculty development in higher education is more robust and suggests that sex, age, academic rank, and institutional factors affect attendance, yet conflicting evidence remains. Some studies associate higher attendance rates with female faculty [20], whereas others do not [21]. Academic rank appears impactful, with some research linking higher attendance to senior ranks [22], non-tenured status [23], or a bimodal relationship [23], where attendance peaks among junior and senior faculty. Additionally, certificates and other incentives, personal recognition, dedicated time, leadership support, and institutional culture promote attendance [24, 25].

Knowing the presenter, especially a friend, can influence professional development attendance [26], as can the value of social relationships at these events [27]. Institutional culture also has a measurable influence on engagement, with one year’s attendance rates predicting the next [28]. Attendance barriers may include faculty workload, fatigue, and burnout [25], as well as workshop timing, length, and location; however, other institutional factors (e.g., school size, professional development location, and school leadership recommendations for attendance) showed minimal impact [22]. Although many studies in higher education assess factors affecting faculty attendance or faculty motivations for attendance, studies evaluating documented attendance are lacking.

Teachers at all levels are encouraged to attend professional development. The Teaching and Learning International Survey (TALIS) [29] included 48 countries and demonstrated that 90% of teachers and principals in lower secondary education attended at least one session in the past year. In the Netherlands [30] in supported professional learning communities, teachers had up to a 95% attendance rate, with intrinsic motivation contributing to attendance. Even these international studies are limited to cross-sectional assessments of attendance rather than predictions of who returns for more.

In medical education, there are few, if any, studies looking at demographic factors affecting attendance in faculty development or evaluating factors associated with return for future faculty development through the assessment of actual attendance data. To optimize attendance at the faculty development program of the Uniformed Services University of the Health Sciences (USUHS), we sought to describe how demographic factors of both faculty attendees and instructors as well as workshop-specific factors impacted future attendance by comparing physician faculty who attended faculty development on a single day and those who attended a subsequent workshop on any future day. By developing a predictive model for factors associated with a continued pursuit of faculty development, our study not only informs internal quality improvement efforts by providing targets for organizational change management but also provides a starting point for other institutions to consider how to optimize their own faculty development programs.

## Materials and Methods

### Study Design and Setting

USUHS is a federal medical school located in Bethesda, Maryland, that uses 22 teaching hospitals nationwide for our required clinical clerkships and dozens of sites worldwide for elective clerkships. We have more than 5,500 faculty located at one of the teaching hospitals or the campus itself. We have a balance of uniformed (i.e., Air Force, Army, Coast Guard, Navy, and Public Health Service) and civilian faculty performing clinical care, leadership, military operational, research, and teaching duties. To meet the needs of these distributed teaching faculty, we have a large faculty development program administered through the Office of Faculty Affairs, overseen by the Associate Dean for Faculty Affairs and the Assistant Dean for Faculty Development. Foundational workshops benefiting all faculty, regardless of specialty, are considered part of the Faculty Development Outreach and Certification for the Uniformed Services (FOCUS); these courses are taught by select faculty embedded in teaching hospitals across the nation who have been trained by the Faculty Affairs Deans (henceforth, community-based instructors) [31]. Additional workshops are taught by the Faculty Affairs Deans or other faculty with specific expertise (henceforth, university-based instructors) at locations throughout the enterprise.

At the time of this study, faculty could earn up to four faculty development certificates: teaching, advanced teaching, academic leadership, and scholarship/research. Earning a certificate requires 30 synchronous credit hours in track-specific workshops, and the advanced teaching certificate requires an additional 30 teaching-track credit hours, three peer observations of teaching, and a reflective writing on those teaching experiences. Our program delivers approximately 600 workshops annually. Each teaching hospital and their more than 200 affiliated ACGME programs may have additional faculty development offerings outside of the university program; these opportunities are not captured herein.

A centralized website enables faculty to search, register for, and withdraw from a myriad of offerings. Additionally, it provides enduring access to handouts and enables dissemination of reminder emails and any workshop-specific preparation work. Faculty can self-assess and choose workshops that apply to their individual needs and interests. Some sets of workshops are “by invitation only” for designated faculty in certain educational roles and require attendance at the full set. For example, the Academic Leadership Meeting is a biannual, invitation-only, week-long set of workshops for Graduate Medical Education (GME) Program Directors.

Website data reports allow programmatic review of the total number of attendees and information about the attendees, instructors, and workshops. Attendee demographics are voluntarily self-reported on their individual registration page on the website.

We conducted a retrospective review of our faculty development website database for physician attendee, instructor, and workshop factors associated with return attendance at faculty development workshops. The primary outcome of our study was to compare those attendees who returned for faculty development on a future day with those who did not return in order to identify factors to target for program improvement.

### Participants

Although our faculty development program started in 2011, numerous changes to standardize the program went into effect by 1 October 2014. As such, we included physician attendees affiliated with School of Medicine clinical departments whose first workshop occurred on/between 1 October 2014 and 30 September 2022 and was taught by a USUHS-affiliated instructor. We followed subsequent workshop attendance through 31 August 2023, allowing at least 11 months of follow-up from the initial workshop attended.

Although instructors affiliated with other institutions taught workshops and we encourage non-physician faculty from allied health professions to attend workshops, we limited our analysis to workshops taught by USUHS faculty development instructors and included only physician attendees (i.e., held the Doctor of Medicine or Doctor of Osteopathic Medicine degree) who were eligible for faculty development certificate credit. GME residents in their final year of training were also included. Additionally, we only assessed workshops for which credit was granted in the certificate program; workshops labeled as “peer observations” were not included. Faculty were eligible to attend workshops on a voluntary basis; an active academic faculty appointment was not required.

### Attendee, Instructor, and Workshop Factors

Physician attendee, instructor, and workshop factors were analyzed based on the first workshop attended. These variables are defined below in the order listed in Table 1 (physician attendee and instructor demographics) and Table 2 (workshop-specific factors).

**Table 1:** Attendee and instructor factors affecting return to faculty development on any future day

	Total *N* = 3213	Returned *N* = 2204 *n* (%)	Did Not Return *N* = 1009 *n* (%)	Absolute Return Ratio	Relative Return Ratio (95% CI)	Adjusted *P*-value
**Attendee Demographics**						
***Attendee Sex ***^***a***^						
Female	884	727 (82.2)	157 (17.8)	4.63	Ref	
Male	1315	1031 (78.4)	284 (21.6)	3.63	0.95 (0.91–0.99)	0.03
***Attendee Race/Ethnicity ***^***b***^						
Asian/Pacific Islander	214	175 (81.8)	39 (18.2)	4.49	0.94 (0.84–1.07)	0.39
Black	61	53 (86.9)	8 (13.1)	6.62	Ref	
Hispanic/Latinx	84	68 (81.0)	16 (19.0)	4.25	0.93 (0.8–1.08)	0.39
Native American	3	3 (100.0)	0 (0)	N/A	N/A	N/A
White	1458	1204 (82.6)	254 (17.4)	4.74	0.95 (0.87–1.06)	0.39
Other	153	119 (77.8)	34 (22.2)	3.5	0.90 (0.79–1.02)	0.39
***Attendee Service ***^***c***^						
Air Force	428	363 (84.8)	65 (15.2)	5.58	Ref	
Civilian	141	111 (78.7)	30 (21.3)	3.70	0.93 (0.84–1.02)	0.35
Army	507	413 (81.5)	94 (18.5)	4.39	0.96 (0.91–1.02)	0.35
Navy	322	266 (82.6)	56 (17.4)	4.75	0.97 (0.91–1.04)	0.56
Public Health Service	5	4 (80.0)	1 (20.0)	4.00	0.94 (0.47–1.22)	0.73
***Attendee Department***						
Medical	2521	1771 (70.2)	750 (29.8)	2.36	Ref	
Surgical	692	433 (62.6)	259 (37.4)	1.67	0.89 (0.84–0.95)	< 0.001
**Instructor Demographics**						
***Instructor Sex***						
Female	2047	1402 (68.5)	645 (31.5)	2.17	0.99 (0.95–1.04)	0.87
Male	1166	802 (68.8)	364 (31.2)	2.20	Ref	
***Instructor Race/Ethnicity ***^***d***^						
Asian/Pacific Islander	176	120 (68.2)	56 (31.8)	2.14	0.82 (0.69–1.0)	0.08
Black	2	2 (100.0)	0 (0)	N/A	N/A	N/A
Hispanic/Latinx	35	29 (82.9)	6 (17.1)	4.83	Ref	
Native American	0	0 (0)	0 (0)	N/A	N/A	N/A
White	2803	1907 (68.0)	896 (32.0)	2.13	0.82 (0.72–0.98)	0.08
Other	18	10 (55.6)	8 (44.4)	1.25	0.67 (0.39–0.97)	0.08
***Instructor Department***						
Basic Science	4	3 (75.0)	1 (25.0)	3.00	0.91 (0.3–1.34)	0.73
Dental	39	32 (82.1)	7 (17.9)	4.57	Ref	
Medical	2565	1755 (68.4)	810 (31.6)	2.17	0.83 (0.73–0.99)	0.1
Surgical	605	414 (68.4)	191 (31.6)	2.17	0.83 (0.72–1.0)	0.1
***Attendee-Instructor Department Dyad***						
Medical-Basic Science	3	2 (66.7)	1 (33.3)	2.00	0.8 (0–1.36)	0.65
Medical-Dental	30	25 (83.3)	5 (16.7)	5.00	Ref	
Medical-Medical	2054	1424 (69.3)	630 (30.7)	2.26	0.83 (0.72–1.0)	0.19
Medical-Surgical	434	320 (73.7)	114 (26.3)	2.81	0.88 (0.76–1.08)	0.38
Surgical-Basic Science	1	1 (100.0)	0 (0)	N/A	N/A	N/A
Surgical-Dental	9	7 (77.8)	2 (22.2)	3.50	0.93 (0.56–1.3)	0.71
Surgical-Medical	511	331 (64.8)	180 (35.2)	1.84	0.78 (0.67–0.95)	0.10
Surgical-Surgical	171	94 (55.0)	77 (45.0)	1.22	0.66 (0.54–0.83)	0.02
***Instructor Position***						
Program Lead	912	635 (69.6)	277 (30.4)	2.29	Ref	
Not Program Lead	2301	1569 (68.2)	732 (31.8)	2.14	0.98 (0.93–1.03)	0.43
***Instructor Type***						
Community-based	1404	934 (66.5)	470 (33.5)	1.99	0.95 (0.90–0.99)	0.03
University-based	1809	1270 (70.2)	539 (29.8)	2.36	Ref	

Data expressed as row %. *P*-value adjusted for within-category multiple comparisons via the Benjamini–Hochberg method

^a^number missing = 1014

^b^number missing = 1240

^c^number missing = 1810

^d^number missing = 179

**Table 2:** Workshop factors affecting return to faculty development on any future day

	Total *N* = 3213	Returned *N* = 2204 *n* (%)	Did Not Return *N* = 1009 *n* (%)	Absolute Return Ratio	Relative Return Ratio (95% CI)	Adjusted *P*-value
***Multiple Instructors***						
Yes	294	203 (69.0)	91 (31.0)	2.23	Ref	
No	3213	2204 (68.6)	1009 (31.4)	2.18	0.99 (0.92–1.08)	0.88
***Cluster Course***						
Yes	257	181 (70.4)	76 (29.6)	2.38	Ref	
No	3250	2226 (68.5)	1024 (31.5)	2.17	0.97 (0.90–1.06)	0.52
***Certificate Track***						
Academic Leadership	449	340 (75.7)	109 (24.3)	3.12	0.98 (0.87–1.11)	0.74
Scholarship/Research	93	72 (77.4)	21 (22.6)	3.43	Ref	
Teaching	2671	1792 (67.1)	879 (32.9)	2.04	0.87 (0.78–0.98)	0.07
***Workshop Location ***^***a***^						
Large Teaching Hospital	2064	1478 (71.6)	586 (28.4)	2.52	Ref	
Medium Teaching Hospital	643	422 (65.6)	221 (34.4)	1.91	0.92 (0.86–0.97)	< 0.01
Small Teaching Hospital	374	214 (57.2)	160 (42.8)	1.34	0.80 (0.73–0.87)	< 0.001
Medical School Campus	132	90 (68.2)	42 (31.8)	2.14	0.95 (0.83–1.06)	0.40
***Workshop Type***						
Conference	68	34 (50.0)	34 (50.0)	1.00	0.64 (0.48–0.79)	< 0.001
Deployed	16	9 (56.25)	7 (43.75)	1.29	0.72 (0.40–1.02)	0.07
Grand Rounds	109	73 (67.0)	36 (33.0)	2.03	0.85 (0.74–0.97)	0.01
Local, Large	994	643 (64.7)	351 (35.3)	1.83	0.82 (0.78–0.87)	< 0.001
Local, Medium	197	135 (68.5)	62 (31.5)	2.18	0.87 (0.79–0.96)	< 0.01
Local, Small	72	53 (73.6)	19 (26.4)	2.79	0.94 (0.80–1.07)	0.39
Roadshow, Large	1057	829 (78.4)	228 (21.6)	3.64	Ref	
Roadshow, Medium	439	281 (64.0)	158 (36.0)	1.78	0.82 (0.76–0.88)	< 0.001
Roadshow, Small	238	130 (54.6)	108 (45.4)	1.20	0.70 (0.61–0.78)	< 0.001
Other	23	17 (73.9)	6 (26.1)	2.83	0.94 (0.71–1.16)	0.59
***Workshop Delivery***						
In-person	2966	2042 (68.8)	924 (31.2)	2.21	0.99 (0.74–1.52)	1.00
Virtual	234	153 (65.4)	81 (34.6)	1.89	0.94 (0.69–1.52)	1.00
Hybrid	13	9 (69.2)	4 (30.8)	2.25	Ref	
***Start Time***						
Morning	2396	1704 (71.1)	692 (28.9)	2.46	Ref	
Afternoon	817	500 (61.2)	317 (38.8)	1.58	0.86 (0.81–0.91)	< 0.001
***Day of Week***						
Monday	164	127 (77.4)	37 (22.6)	3.43	0.97 (0.88–1.05)	0.43
Tuesday	870	697 (80.1)	173 (19.9)	4.03	Ref	
Wednesday	911	663 (72.8)	248 (27.2)	2.67	0.91 (0.86–0.96)	< 0.001
Thursday	787	486 (61.8)	301 (38.2)	1.61	0.77 (0.72–0.82)	< 0.001
Friday	458	219 (47.8)	239 (52.2)	0.92	0.60 (0.54–0.66)	< 0.001
Saturday	11	6 (54.5)	5 (45.5)	1.20	0.68 (0.33–1.03)	0.08
Sunday	12	6 (50.0)	6 (50.0)	1.00	0.62 (0.31–0.96)	0.04
***Month of Year***						
July	59	51 (86.4)	8 (13.6)	6.38	Ref	
August	96	75 (78.1)	21 (21.9)	3.57	0.90 (0.78–1.05)	0.20
September	572	403 (70.5)	169 (29.5)	2.38	0.82 (0.73–0.92)	0.01
October	205	149 (72.7)	56 (27.3)	2.66	0.84 (0.74–0.97)	0.03
November	230	162 (70.4)	68 (29.6)	2.38	0.81 (0.72–0.93)	0.01
December	170	113 (66.5)	57 (33.5)	1.98	0.77 (0.66–0.89)	< 0.01
January	339	210 (61.9)	129 (38.1)	1.63	0.72 (0.63–0.82)	< 0.001
February	467	332 (71.1)	135 (28.9)	2.46	0.82 (0.74–0.94)	0.01
March	351	235 (67.0)	116 (33.0)	2.03	0.77 (0.69–0.89)	< 0.01
April	364	255 (70.1)	109 (29.9)	2.34	0.81 (0.72–0.93)	0.01
May	283	183 (64.7)	100 (35.3)	1.83	0.75 (0.66–0.86)	< 0.01
June	77	36 (46.8)	41 (53.2)	0.88	0.54 (0.40–0.69)	< 0.001

Data expressed as row %. *P*-value adjusted for within-category multiple comparisons via the Benjamini–Hochberg method

^a^Workshops delivered at large teaching hospitals included *local, large*; *roadshow, large*; and 13 *conference* workshops

Workshops delivered at medium teaching hospitals included *local, medium*; *roadshow, medium*; and 7 *conference workshops*

Workshops delivered at small teaching hospitals included *local, small*; *roadshow, small*; 68 *conference*; and all *deployed* workshops

Workshops delivered at the medical school campus included *grand rounds* and *other* workshops

For simplicity, physician attendees will be referred to as *physicians* or *attendees*; those delivering faculty development will be referred to as *instructors*. Demographic variables analyzed included self-reported sex, race/ethnicity, Uniformed Service affiliation, and departmental affiliation. Instructor affiliation with a particular Uniformed Service was not measured, as some instructors retired from active duty during the study period and had ongoing program engagement, confounding their affiliation.

All attendees and instructors were categorized according to their self-reported primary departmental affiliation. Those assigned to the Departments of Anesthesia, Gynecologic Surgery & Obstetrics, and Surgery were designated *surgical*. Those assigned to the Departments of Dermatology, Family Medicine, Medicine, Military & Emergency Medicine, Neurology, Pathology, Pediatrics, Physical Medicine & Rehabilitation, Preventive Medicine & Biostatistics, Psychiatry, and Radiology & Radiological Sciences were designated *medical*. Instructors assigned to the Departments of Biochemistry or the Learning Resource Center were designated *basic science.* Instructors affiliated with the university’s Postgraduate Dental College were designated *dental*. We assessed departmental affiliation concordance between attendees and instructors for the first workshop attended using an *attendee-instructor dyad* variable that captured all observed pairings.

We dichotomized the *position* of instructors within the faculty development program into *program lead* (i.e., the Associate Dean for Faculty Affairs) vs *not program lead* categories. This distinction was made because the program lead delivered more workshops than any other instructor and had advanced experience in leading faculty development. Instructors were further categorized by an *instructor type* variable distinguishing between *community-based* vs *university-based instructors*. As a rule, community-based instructors were specifically trained to facilitate faculty development and deliver a subset of up to 22 foundational workshops listed in Supplemental Table 1, constituting the aforementioned FOCUS program. University-based instructors were based at the medical school campus and, as a group, covered an expanded set of topics beyond those foundational workshops, numbering > 150 topics and exploring both those foundational and more nuanced or niche areas within medical education.

Multiple workshop-specific factors were also considered. To optimize program planning, workshops were assessed according to the number of instructors delivering them: either a single instructor or multiple instructors. Workshops designated *cluster courses* were standard “sets of workshops” that were 1–5 days long, taught by multiple instructors, and required attendance at the full course. We collapsed each cluster course into a single course so that required attendance within that cluster would not confound our primary outcome of return on a future day. Workshops taught by multiple instructors, including cluster courses, were only included in the analysis where specified in Table 2, as the presence of multiple instructors, topics, and times would confound the analysis of many instructor- and workshop-specific factors.

Workshops were categorized into one of three *certificate tracks*: teaching, academic leadership, and scholarship/research. Workshops were further characterized by the *location* at which they were given: on the medical school campus or at a teaching hospital, stratified by institutional size. Teaching hospitals were designated *small*, *medium*, or *large* based on the number of ACGME training programs they housed: hospitals with 1–2 programs were *small*; 3–10 were *medium*; and > 10 were *large*. The variable *workshop type* was considered based on the educational context within which it was given. Some workshops were given at specialty conferences, in military deployed locations, or grand rounds, and also categorized based on size, as noted in Table 2. *Local* workshops were delivered by community-based instructors located at the various teaching hospitals, whereas *roadshows* involved a traveling group of university-based instructors who came to the teaching hospitals in an outreach effort. Both local and roadshow workshops were stratified by the size of the teaching hospital at which they were given. Workshops were also considered based on the medium of delivery (i.e., *in-person*, *virtual*, or *hybrid*) and the time, day, and month of delivery.

### Statistical Methods

We employed descriptive statistics to assess demographic and workshop factors. To answer the question of what factors independently influenced attendees to return on any future day, we designed a multivariate model to determine a risk ratio (i.e., *return ratio*). Each factor’s *absolute return ratio* was calculated by dividing the number of attendees who returned on a subsequent day by the number who did not return. The *relative return ratio (RRR)* was calculated via bootstrapped logistic regression, with the categorical reference set to the variable with the highest absolute return ratio. We conducted these analyses using the *R* statistical language (v4.4.1; R Core Team, 2024 on Windows 11 × 64 (build 22,631)) [32]. We formatted these data and visualized the results with the *tidyverse* package (v.2.0.0) [33]. We calculated model-based risk ratios and 95% confidence intervals via bootstrap [34] with the *epitools* package (v.0.5–10.1) [35]. Statistical significance was set at *p* ≤ 0.05, adjusted for within-category multiple comparisons using the Benjamini–Hochberg method. We approached missing data using listwise deletion such that only observations with complete data were included in the multivariate logistic regression model.

### Ethics

This retrospective review was conducted as part of the program evaluation for faculty development. The protocol, DBS.2022.371, was reviewed by the USUHS Human Research Protection Program and determined to be p*rogram evaluation*, exempt from review by the Institutional Review Board.

## Results

### Program Overview

**Fig. 1:**
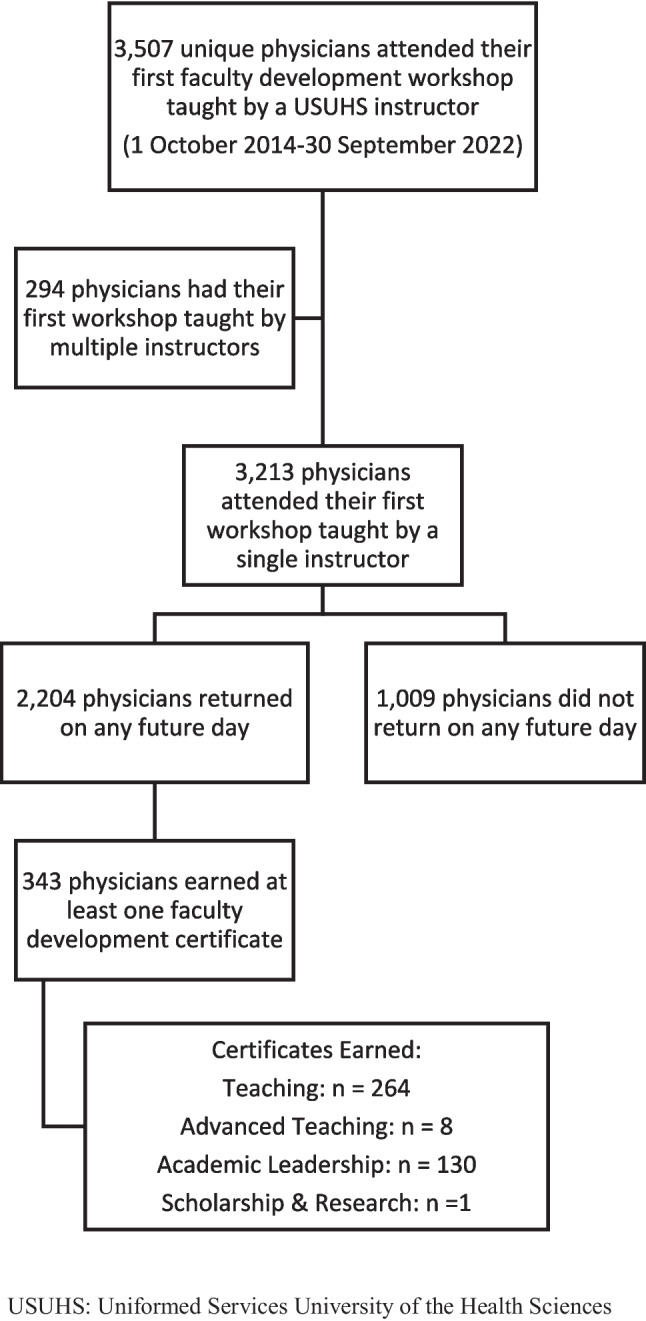
Flow of attendees in the analysis of factors affecting return to faculty development on any future day. USUHS: Uniformed Services University of the Health Sciences

Between 1 October 2014 and 30 September 2022, 3,507 unique physicians attended their first faculty development workshop taught by a USUHS instructor. Of those, 3,213 (91.6%) attended a first workshop taught by a single instructor (Fig. 1; Tables 1–2). Of first-time attendees with a single instructor, 2,204 (68.6%) returned for a workshop on any future day, and 343 (10.7%) earned at least one certificate: 264 teaching, 8 advanced teaching, 130 academic leadership, and 1 scholarship/research. Among those who returned on any future day, 966/2,204 (43.8%) returned within one week of their first class, of whom 886/966 (91.7%) returned the next calendar day; by contrast, 528/2,204 (24.0%) first returned a year or more later (Table 3).

**Table 3:** Number of days from first day to next day attended

Number of days from first day to next day attended	Number of Attendees (*N* = 2,204) n (%)
1 day	886 (40.2)
2–7 days	80 (3.6)
8–14 days	35 (1.6)
15 days to < 6 months	463 (21.0)
6 months to < 1 year	212 (9.6)
1 year to < 2 years	274 (12.4)
≥ 2 years	254 (11.5)

Table 1 presents demographic data. Females comprised 40.2% of attendees reporting sex vs 59.8% male; 73.9% identified as white, 10.8% as Asian/Pacific Islander, 4.3% as Hispanic, and 3.1% as black. The vast majority (90.0%) were uniformed, with 36.1% serving in the Army, 30.5% in the Air Force, and 23.0% in the Navy. Most (78.5%) physician attendees were affiliated with medical departments compared to 21.5% surgical. It is notable that 79.8% of physicians had a first instructor affiliated with a medical department, 63.7% had a female instructor, 92.4% had a white instructor, and 28.4% had the Associate Dean for Faculty Affairs (a white female from a medical department). First workshops showed concordance between departmental classifications of the attendee and instructor 69.2% of the time (e.g., both members of the attendee-instructor dyad were assigned to a medical department or both to a surgical department).

Of the 3,213 first workshops considered, most were delivered by university-based instructors (56.3%), fell within the teaching track (83.1%), and were standalone, non-cluster workshops (92.7%, Tables 1–2). Roadshows accounted for 54.0% of first workshops, and 64.3% were conducted at large teaching hospitals. Only 7.7% of workshops involved some degree of virtual delivery. Less than 1% of workshops were offered on weekends, and the frequency of first workshops was highest in the middle of the week (Tuesday-Thursday). Workshops were offered each month of the year, but the nadir occurred in the summer (June–August). Three-quarters (74.6%) of first workshops occurred in the morning.

### Modeling

**Fig. 2:**
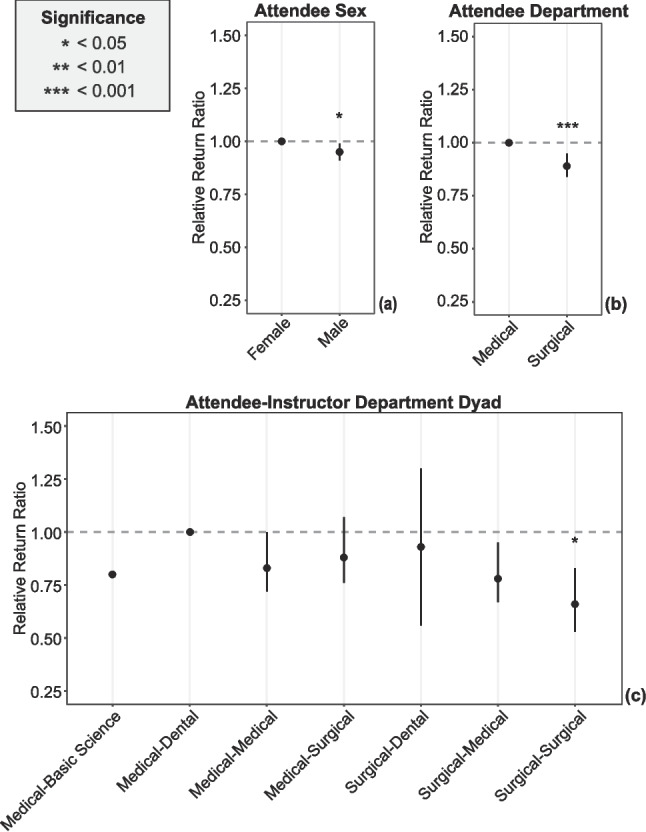

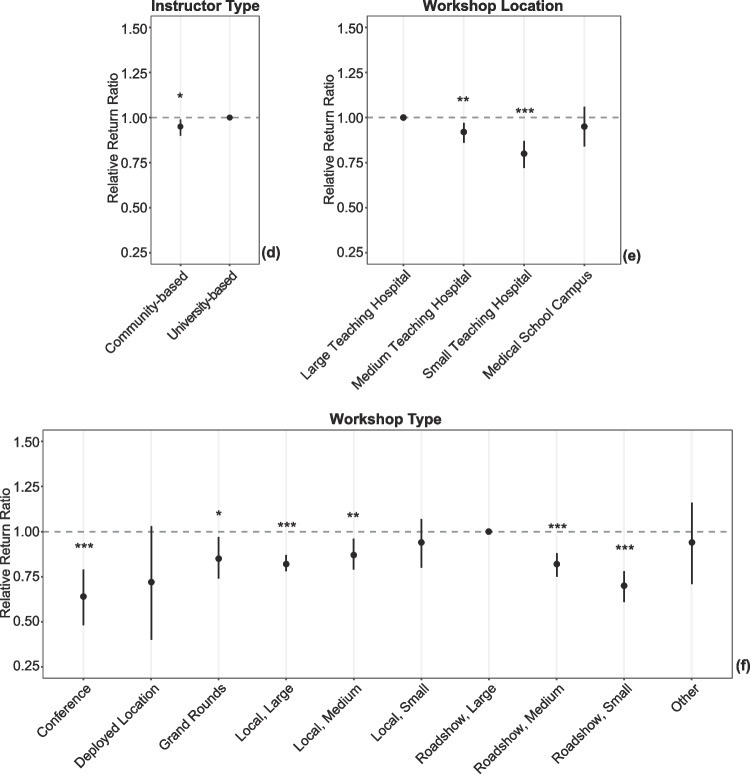

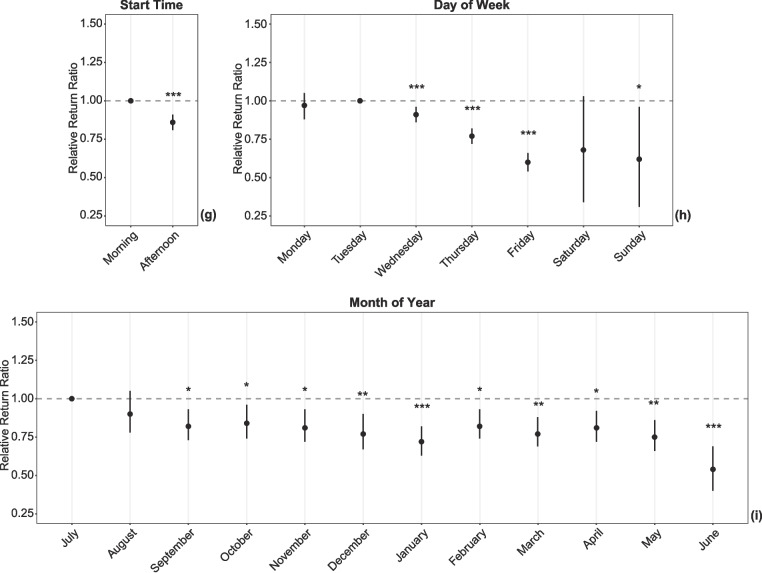
Relative return ratio for factors associated with return to faculty development on any future day. Each plot demonstrates the relative return ratio (RRR) for factors associated with the return of physician faculty for faculty development on any future day compared to those who attended on only a single day. The RRR is a risk ratio calculated via bootstrapped logistic regression, with the categorical reference set to the variable with the highest absolute return ratio. Error bars demonstrate the 95% confidence interval. Asterisks indicate statistical significance: * for *p* < 0.05, ** for *p* < 0.01, and *** for *p* < 0.001. **a** RRR for attendee sex. **b** RRR for attendee departmental affiliation. **c** RRR for the dyad juxtaposing the attendee’s and their first instructor’s departmental affiliation, respectively. **d** RRR for the instructor type for the first workshop. **e** RRR for the first workshop’s location. **f** RRR for the workshop type of the first workshop. **g** RRR for the first workshop’s start time. **h** RRR for the day of the week of the first workshop. **i** RRR for the month of the year of the first workshop

Using a multivariate model, we determined a relative return ratio to assess which attendee, instructor, and workshop factors were independently associated with physician return on any future day (Tables 1–2, Fig. 2). For attendee factors, males were 5% less likely to return than their female counterparts (RRR 0.95; 95% CI 0.91 to 0.99; Fig. 2a). Those affiliated with a surgical department were 11% less likely to return than their medical counterparts (RRR 0.89; 95% CI 0.84 to 0.95; Fig. 2b). For instructor factors, there was a notable interaction when considering the attendee-instructor dyad in the first workshop (Fig. 2c). When both the attendee and instructor were affiliated with a surgical department, attendees were 34% less likely to return on a future day compared to the reference standard of the medical-dental dyad (RRR 0.66; 95% CI 0.54 to 0.83); no other pairing reached statistical significance. Additionally, attendees were 5% less likely to return when their first workshop was delivered by a community-based vs a university-based instructor (RRR 0.95; 95% CI 0.90 to 0.99; Table 1, Fig. 2d).

There were a number of significant workshop factors. Compared to physicians attending their first workshop at a large teaching hospital (Fig. 2e), physicians were 8% less likely to return when attending their first workshop at a medium-sized hospital (RRR 0.92; 95% CI 0.86 to 0.97) and 20% less likely to return at a small hospital (RRR 0.80; 95% CI 0.73 to 0.87). A similar pattern held true when their first workshop was part of a roadshow at a medium-sized hospital (RRR 0.82; 95% CI 0.76 to 0.88; Fig. 2f) or a small hospital (RRR 0.70; 95% CI 0.61 to 0.78) instead of a large hospital. In contrast, when the first workshop was a local workshop conducted by community-based instructors, a reduced return ratio was seen for medium-sized (RRR 0.87; 95% CI 0.79 to 0.96) and large hospitals (RRR 0.82; 95% CI 0.78 to 0.87), but not small hospitals. When first workshops were conducted as grand rounds or at a military specialty conference, there was also a significantly reduced likelihood of physicians returning on a subsequent day.

Physicians attending first workshops in the afternoon showed a 14% decrease in the likelihood of return (RRR 0.86; 95% CI 0.81 to 0.91; Fig. 2g). Compared to a reference standard of first workshops occurring on Tuesday, physicians attending their first workshop later in the week were less likely to return, with decreases of 23–40% for first workshops on Thursday through Sunday (Table 2, Fig. 2h). The reduced likelihood for first workshops on Wednesdays was statistically significant but smaller in magnitude. Though Saturday was not associated with a statistically significant reduction, the magnitude was large, and there was a trend towards reduction; the lack of significance may be impacted by Saturday’s small sample size. While first workshops occurring in July were associated with a greater likelihood of return compared to any other month but August (Fig. 2i), first workshops occurring in June were associated with the greatest reduction (46%) in the likelihood of return (RRR 0.54; 95% CI 0.4 to 0.69).

## Discussion

Engagement in faculty development is crucial for enhancing teaching quality and fostering career advancement. We evaluated factors that could be influenced by the leadership of a faculty development program to enhance engagement, including when faculty development is delivered, by whom, and how. Other research identifies individual and institutional barriers affecting attendance, including lack of time, insufficient institutional support, and inflexible programming [36]. Our study advances current knowledge on the barriers and facilitators of faculty development attendance by applying a model that assesses the likelihood of return for future faculty development workshops based on attendee, instructor, and workshop factors. In addressing the challenges and solutions to enhance faculty development participation, van Bruggen and colleagues [36] proposed the “4 Cs” of faculty development: Competence, Context, Community, and Career. This framework provides a comprehensive approach to understanding faculty needs and how they can be supported to encourage continued professional development participation. We apply the 4 C framework to our faculty development return rates, offering insights into overcoming barriers and improving future faculty engagement.

### Competence

Competence refers to the skills faculty need to fulfill their teaching responsibilities [36]. Previous data suggest that offering a broad range of topics and providing flexible attendance options contribute to faculty’s perceived competence and their likelihood of return [11]. In our cohort, workshops delivered by university-based instructors were associated with a higher return rate compared to those delivered by community-based instructors. We did not assess differences in the quality of teaching or facilitation skills between community-based and university-based instructors, though community-based instructors went through a rigorous training process that incorporated a deliberate practice model involving multiple observed teaching encounters. We posit that because community-based instructors had a more limited set of topics on which they were trained, the higher return rate for university-based instructors may reflect a desire for novelty or greater breadth and depth of topics after self-assessed mastery of core topics. Alternatively, the difference in return rate may correspond to a “roadshow” effect at our geographically dispersed teaching hospitals, as university-based instructors traveled from the main campus, often in groups, to deliver workshops to which attendees otherwise had limited access. This association is strengthened by a significantly lower return rate when the first workshop was delivered by community-based instructors at larger hospitals, whose sessions were enriched for the core topics in Supplemental Table 1. While the return rate was lower for community-based instructors compared to university-based instructors, it is notable that the absolute difference in the proportion of returnees was small (local large, 64.7% vs roadshow large, 78.4%; community-based, 66.5% vs university-based, 70.2%), suggesting the importance of having a multifaceted program adaptable to diverse faculty needs.

The competence domain may impact how other schools engage with teaching hospitals or community faculty and encourage the development of additional, novel programming based on a needs assessment. Flexible programming with varied content can help address competence needs by offering faculty access to tools and knowledge that may be changing regularly (e.g., technology integration in teaching, reflective practice, and competency-based education). Workshops that move beyond foundational topics (e.g., feedback, learning climate, and direct observation) and cater to a broader range of interests may increase the intrinsic motivation described in prior research, fostering continued attendance [30].

### Context

Context includes the resources faculty need to pursue professional development (e.g., time, funding, staff support, and facilities) [36]. Steinert noted that faculty development programs often neglect critical environmental factors [10], and scheduling has been identified as a challenge even outside of medical educaiton [27]. Our findings suggest that structural program elements influence faculty’s likelihood of return, as the return ratio was significantly associated with the day, week, and time of workshop delivery, though program design influenced this. We often offered workshops several days in a row, especially at large hospitals, as evidenced by nearly half of returnees doing so within a week of their first workshop. We also offered more workshops in the morning and scheduled them according to the GME calendar life cycle. While these may imply a confounding by indication, they suggest that other schools should consider the unique context of their clinician-educator faculty at teaching hospitals and community sites in designing accessible faculty development schedules. Scheduling mismatches with clinical, research, and administrative workflow may adversely affect continued faculty engagement [12, 22]. While institutional support of faculty development via scheduling considerations was important within the Military Health System where reimbursement for providers is not tied to clinical productivity, heightened institutional attention to context and engagement of clinical/administrative leadership for protected time is critical in the civilian sector, even globally [15, 37].

Our program’s geographic scope is a unique contextual feature; however, it is applicable to how medical schools engage with faculty embedded in regional medical campuses or community partnerships. Our data highlight improved return rates when outreach is accomplished and prioritized, as demonstrated by higher return for roadshows at large teaching hospitals compared to other workshop types. This higher return ratio may reflect a combination of factors: strong leadership buy-in, advanced planning, and the availability of outside experts offering novel content, reflecting a coordinated “wish list” from on-site faculty. These roadshows often span multiple days with multiple concurrent workshops, allowing faculty to return for additional sessions within the same visit. By contrast, program offerings at smaller hospitals are typically one-day events with a less robust schedule, and those facilities may not have trained community-based instructors, creating an opportunity differential. When a program sends its representatives to visit faculty at their work sites rather than requiring faculty to travel to the main campus, it may minimize structural, time, and location barriers.

Context also includes the delivery method. Our study only included two years of virtual programming beginning with the pandemic in 2020, which may have influenced our ability to detect a difference between delivery methods. However, virtual options may provide an opportunity to engage with smaller teaching sites and enable faculty to share experiences and tips across sites and specialties.

Together, these contextual features may influence how schools develop and resource their faculty development programs, as they underscore the importance of considering logistical factors and community outreach in designing programs to decrease attendance barriers and increase faculty engagement.

### Community

The community aspect of faculty development emphasizes the creation of supportive networks for collaboration, mentoring, and advocacy [36]. Our findings reveal some disparities in return rates among different faculty groups, particularly surgeons. Surgeons were less likely to return on subsequent days, especially when the first workshop’s attendee and instructor were both from surgical departments, though the numbers in the surgical-surgical dyad were small (5.3% of first workshops). This lower return rate was unexpected, as we had hypothesized that shared professional backgrounds between attendee and instructor would foster stronger connections and encourage further attendance. We propose several hypotheses for this lower return rate for surgeons. First, clinical demands (e.g., operating room schedules), may limit surgeons’ availability for repeated participation due to their unique career field. Institutional or departmental factors, such as lack of operating room time and challenges in case distribution and scheduling, are known to adversely affect clinical practice development in academic surgeons [38]. Second, workshop content may not have been perceived as directly relevant to surgical practice, even when the instructor was also a surgeon. Indeed, surgeons may have preferentially attended the workshop they perceived as most relevant and not returned for other workshops perceived as less relevant. A survey of US surgical educators found that intraoperative teaching skills was a top priority for surgical faculty development [37], a topic for which we had limited specific content.

Faculty development program leaders could implement a change management framework to increase community engagement [25]. For example, schools could design their faculty development programs by placing a focus on surgeon engagement through conducting a needs assessment, increasing surgical leadership buy-in, and expanding the number of surgeon instructors, not only to serve as peer role models but also to enhance workshop relevance [37]. Future studies could consider a qualitative exploration of surgeons’ perceptions of faculty development through interviews to better understand the barriers they face and their motivations for attendance. Building a stronger community of practice within surgery may be key to increasing engagement.

### Career

The final “C,” career, focuses on ensuring the career viability and vitality of faculty by recognizing and rewarding teaching excellence, creating clear advancement pathways, and providing opportunities for professional growth [36]. Our program highlights the importance of institutional support in this domain, as roadshows at large hospitals—where local leadership actively promoted the sessions and the university provided advanced scheduling—had the highest return rates. The ceremonial awarding of certificates to participants further reinforced the institutional value placed on faculty development; incentivizing faculty development through awards has been shown to be important in higher learning [24–26]. Thus, considering how to optimally incentivize faculty development as a pathway to career progression is important in designing programs within the institutional and community context.

## Limitations & Strengths

Beyond the standard limitations of a retrospective analysis, including an inability to determine causality, our study has several limitations. First, attendees were only tracked within our system when they attended a workshop; therefore, we were unable to determine whether those who did not return had an ongoing affiliation with our organization or an opportunity for subsequent attendance. That said, even though a plurality of attendees returned within a week of their first workshop, nearly one-quarter returned a year or more later. The reason for this delayed return is not clear. It may correspond to the annual faculty development requirements for GME core faculty, long-term interest in faculty development without an opportunity for interim attendance, or the strength of our longitudinal, large dataset to capture attendance patterns over time. Second, although we know that more than two-thirds of our faculty were Assistant Professors, our data did not allow assessment of the impact of attendee academic rank, teaching experience, or academic role (e.g., core faculty, program director, clerkship director, etc.) on the likelihood of return. This variable has been considered in other studies of professional development engagement in higher learning [21, 22] and could be an opportunity for enhanced data collection for our future attendees. Third, we had a large amount of missing data for several demographic variables since these were voluntarily self-reported (i.e., attendee service affiliation and sex, and attendee and instructor race/ethnicity), limiting our ability to detect a difference. Fourth, despite assessing a longitudinal system-wide cohort, several categories comprised small sample sizes, and the effect sizes were generally small. Nonetheless, the findings provide insight into potential targets for quality improvement efforts to increase the return rate for future faculty development. Fifth, we were unable to determine whether attendees received faculty development through alternate sources (e.g., non-university resources within the hospital or local civilian partner institutions). This unmeasured confounder could have falsely increased the difference between groups if our non-returning faculty were actually attending outside faculty development opportunities. Sixth, given the number of variables assessed, there is a risk of multicollinearity; however, a correlation analysis revealed that our data were robust to determine independent factors associated with return attendance.

A major strength of this study is its inclusion of system-wide data for a large, geographically dispersed healthcare system with thousands of faculty supported by a faculty development program with a large number of workshop offerings. While our study may not generalize to a single-site institution, our study applies to medical schools that increasingly rely on community partners and regional campuses for student clinical education. Our findings shed light on a far-reaching, successful, and predominately in-person faculty development program that overcame the logistical challenges of its geographic scope, elucidating opportunities for continued quality improvement as institutions consider when, by whom, and how faculty development can be delivered to enhance return. Future analyses could include qualitative or mixed methods studies assessing motivation to return for future faculty development or evaluating the impact of instructor experience as an educator or faculty development facilitator to further refine the program and optimize teaching across sites.

## Conclusion

Faculty development programs must address individual and institutional barriers to faculty engagement. Our data suggest that there are demographic and workshop factors that can influence continued attendance in professional development offerings. During program evaluation of faculty development programs, leaders should consider attendance patterns related to faculty competence, context, community, and career to meet the dynamic needs of their faculty.

## Supplementary Information

Below is the link to the electronic supplementary material.

Supplementary file 1 (DOCX 15.0 KB)

## Data Availability

The datasets generated and/or analyzed during the current study are available from the corresponding author on reasonable request.

## Declarations

### Disclaimer

The opinions and assertions expressed herein are those of the author(s) and do not reflect the official policy or position of the Uniformed Services University of the Health Sciences, the Defense Health Agency, the Department of Defense, or the U.S. Government. Mentions of trade names, commercial products, or organizations does not imply endorsement by the U.S. Government.

Some authors are either military servicemembers (DAL, DB, DH, and GHR) or employees of the US Government (JS, TM, SS, BZ). This work was prepared as part of their official duties. Title 17 U.S.C. 105 provides that “Copyright protection under this title is not available for any work of the United States Government.” Title 17 U.S.C. 101 defines a United States Government work as a work prepared by a military servicemember or employee of the United States Government as part of that person’s official duties.
