# Supplementary material for: What Keeps Faculty Coming Back? Factors Associated with Continued Pursuit of Faculty Development
Source: Med Sci Educ. 2025 Jun 21;35(4):2109–22. doi: 10.1007/s40670-025-02422-8 (PMC12532536; doi:10.1007/s40670-025-02422-8)
Supplement: Supplementary file 1 — (DOCX 15.0 KB) [file 40670_2025_2422_MOESM1_ESM.docx]

**Supplemental Information for:**

**What Keeps Faculty Coming Back?**

**Factors Associated with Continued Pursuit of Faculty Development**

**Journal:** *Medical Science Educator*

**Authors:** David A. Lindholm, MD; Jessica Servey, MD; Rhiana Saunders, MD; Thomas McFate, PhD; Salvatore Sidoti, PhD; Bizualem Zelelew, PhD; Dana Blyth, MD; Diane Hale, MD; Gayle Haischer-Rollo, MD

**Corresponding Author:** David A. Lindholm, MD (ORCID: 0000-0001-5428-7404)

**Corresponding Author Affiliations:** Uniformed Services University of the Health Sciences, Bethesda, MD, USA & Brooke Army Medical Center, Joint Base San Antonio-Ft Sam Houston, TX, USA

**Corresponding Author E-mail Address:** david.lindholm@usuhs.edu

**-----------------------------------------------------------------------------------------**

**Supplemental Table 1: Faculty Development Workshops on which Community-based Instructors are Trained**

Bedside Teaching
Challenges in Transitions of Care
Feedback
Large Group Teaching
Cutting Edge Feedback: Framework for Feedback for Surgical Procedure
Precepting
Learning Climate
Small Group Teaching: From Loss of Control to Total Group Learning
Narrative Assessments
Beyond Average: Pushing Learners to Greatness
Direct Observation
Mentoring, Coaching, and Sponsoring
Role Modeling
Debriefing in Medical Simulation
Creating a Wellness Curriculum
Clinical Goal Setting
Active Learning
Teaching Procedures
Supervision
Nighttime Education
Milestones and EPAs: Feeding the CCC
Poster Creation
